# Short-Term Prognostic Index for Breast Cancer: NPI or Lpi

**DOI:** 10.4061/2011/918408

**Published:** 2010-12-28

**Authors:** V. Van Belle, J. Decock, W. Hendrickx, O. Brouckaert, S. Pintens, P. Moerman, H. Wildiers, R. Paridaens, M. R. Christiaens, S. Van Huffel, P. Neven

**Affiliations:** ^1^Division SCD, Department of Electrical Engineering (ESAT), K.U.Leuven, Kasteelpark Arenberg 10/2446, 3000 Leuven, Belgium; ^2^School of Biological Sciences, University of East Anglia (UEA), NR4 7TJ Norwich, UK; ^3^School of Medicine, Health Policy and Practice, University of East Anglia, NR4 7TJ Norwich, UK; ^4^Multidisciplinary Breast Centre (MBC), University Hospitals Leuven, Leuven, Belgium; ^5^Department of Pathology, University Hospitals Leuven, Leuven, Belgium; ^6^Department of General Medical Oncology, University Hospitals Leuven, Leuven, Belgium; ^7^Department of Gynaecological Oncology, University Hospitals Leuven, Leuven, Belgium

## Abstract

Axillary lymph node involvement is an important prognostic factor for breast cancer survival but is confounded by the number of nodes examined. We compare the performance of the log odds prognostic index (Lpi), using a ratio of the positive versus negative lymph nodes, with the Nottingham Prognostic Index (NPI) for short-term breast cancer specific disease free survival. A total of 1818 operable breast cancer patients treated in the University Hospital of Leuven between 2000 and 2005 were included. The performance of the NPI and Lpi were compared on two levels: calibration and discrimination. The latter was evaluated using the concordance index (cindex), the number of patients in the extreme groups, and difference in event rates between these. The NPI had a significant higher cindex, but a significant lower percentage of patients in the extreme risk groups. After updating both indices, no significant differences between NPI and Lpi were noted.

## 1. Introduction

In women with an operable breast cancer, the lymph node status is amongst the most important prognostic parameters for disease free survival (DFS). An operable breast cancer patient was defined as “any consecutive patient with an invasive breast cancer without a contraindication for primary surgery”. This excludes patients with metastatic disease and those previously treated for breast cancer such as those receiving neoadjuvant systemic treatment. A positive axillary lymph node status is associated with a clear increase in risk of recurrence and mortality. Moreover, patients with at least four positive lymph nodes have a worse prognosis compared with those with three or less positive nodes [1]. Examination of axillary lymph nodes for tumor involvement can be performed by axillary lymph node dissection or sentinel lymph node biopsy [2]. Sentinel lymph node biopsy enables an accurate nodal staging by examination of one or a few sentinel lymph nodes, while obviating invasive surgery of axillary dissection [3]. In case the sentinel lymph node is positive, a complete axillary lymph node dissection is performed [4]. However, guidelines defining the minimum number of lymph nodes to examine in axillary lymph node dissection and/or sentinel lymph node biopsy are not available. As the likelihood of finding positive nodes in the axilla increases with the number of nodes removed during axillary lymph node dissection an increasing number of studies is examining the prognostic value of the nodal ratio, which were nicely reviewed by Woodward and coworkers [5]. 

Furthermore, the established Nottingham Prognostic Index (NPI) [6] is put under debate as it takes only the number of positive lymph nodes into consideration. Vinh-Hung and colleagues introduced a ratio-based nodal prognostic index based on the empirical log odds of nodal involvement [7, 8]. Similar to the NPI, this log odds prognostic index (Lpi) is computed based on the tumor size, histological grade, and axillary nodal involvement. The latter considering the number of negative nodes besides the number of positive lymph nodes. Using the Surveillance, Epidemiology and End Results (SEER release 2005, *n* = 7526) public database, they compared the established NPI with the alternative Lpi prognostic index. They reported a better prognostic separation with regard to overall survival as well as breast cancer specific survival between risk groups when defined by the Lpi [7]. This study aimed at evaluating the prognostic value of Lpi in a dataset of 1818 operable breast cancer patients from our institution.

## 2. Materials and Methods

A total of 2,024 operable female breast cancer patients who were treated in the University Hospitals of Leuven between 2000 and 2005 and underwent lymph node dissection were available for this study. Only patients without prior pathologic sentinel lymph node evaluation (*n* = 1,838) were included. After exclusion of 20 patients lacking information on size, grade, or lymph node status, 1,818 patients remained for analysis. None of these patients received neoadjuvant therapy. Tumor characteristics and lymph node status were retrieved from pathology reports and together with clinical data gathered in our central breast cancer database. The lymph node status was defined according to AJCC criteria. Isolated tumor cells were considered as lymph node negative. Surgical treatment consisted in wide local excision plus axillary dissection followed by whole breast radiotherapy plus a boost on the tumour bed, or modified radical mastectomy when breast-conserving surgery was not indicated. Chest wall RT following mastectomy was given to patients with T3 or T4 tumors, with positive lymph nodes or with positive tumour section margins. Irradiation to the internal mammary chain was performed only in cases of axillary lymph node involvement or medial tumour sites. Endocrine therapy (HT) was prescribed if the expression ER and/or PR were present. Although tamoxifen 20 mg/day for five years was the standard HT, many postmenopausal women also received an oral aromatase-inhibitor for a period of five years. Anthracycline-based chemotherapy (CT) was given if patients were classified as intermediate or high risk for relapse but endocrine sensitivity, NPI, and age at diagnosis were also important when deciding upon systemic adjuvant therapy.

Patients were followed until June 2009 for cancer recurrence through clinical records or through contact with the general practitioner by phone. Median follow-up time was 5.92 year (interquartile range (IQR): 4.33–7.12 year). During the follow-up period, 67 patients developed local recurrence and 193 developed distant metastasis. For patients with bilateral cancer at first diagnosis, the worse NPI and concordant Lpi value were taken into account.

NPI and Lpi were calculated using tumor size, histological grade, number of positive and/or negative lymph nodes. Tumor size was defined as the maximum diameter of the tumor in cm. Histological grading of tumors was performed according to the Ellis and Elston system [9]. The NPI was computed as 0,2xtumor size (cm) + grade(1–3) + nodal score. The nodal score was defined (1) when no nodal involvement was present, (2) when ≤3 positive lymph nodes, and (3) when >3 positive lymph nodes were present. The Lpi was computed using an equation published in [8]: size(cm) + 1  if  grade > 2(0  otherwise) + log ((npos + 0.5)/(nneg + 0.5)), where npos, en, and nneg are the number of positive and negative nodes, respectively.

## 3. Statistical Analyses

Patients were followed from the date of surgery until a breast cancer-related event (locoregional recurrence or distant metastasis) occurred. In case no event was observed within the study time, patients were censored at the last date of followup. This time will be denoted as the disease-free survival time (DFS). Survival curves are calculated by means of the Kaplan-Meier (KM) method. The logrank test is used to test for statistical significant differences in survival. All variables used in both prognostic models were used in a univariate Cox model to check their relevance on our dataset. The prognostic models are validated on two levels: discrimination and calibration. The discrimination ability is summarized in the concordance index (cindex) [10]. For clinical practice, a model categorizing patients in the most extreme risk groups is preferred [11]. Therefore, the percentage of patients in the low and high risk groups are calculated (EXT%). Additionally, the difference in event rates between high and low risk groups are reported (EvR). The event rate is calculated as the number of events divided by the total followup of all patients in the group. The inverse of the event rate is interpreted as the number of years one has to wait before an event is expected to occur. Bootstrap adjusted 95% confidence limits were calculated on all validation measures, using 1000 bootstrap samples of the dataset. Calibration plots are made to check whether the predicted survival chance corresponds with the true survival. Therefore, the test data were divided into five groups according to the value of the NPI (Lpi). For each group, the observed survival as calculated by the Kaplan-Meier estimator at 5 years and the median predicted survival at 5 years are calculated. The calibration plot summarizes the results. However, since the NPI was developed on a dataset containing 351 operable breast cancer patients from 1976–1981 and the Lpi on the SEER 2005 dataset containing 7526 patients from 1988–2004, it is reasonable to assume that these patient populations differ from ours. Not only is the treatment continuously changing, but the number of examined lymph nodes may differ from centre to center. Therefore, our data was randomly divided in a training and test set (both containing 50% of the data). The model predicted survival was obtained from the training set. Calibration was then checked on the test set. 

Steyerberg [12] proposed model updating in cases where it is expected that patient populations between training and test sets will differ. Populations can differ due to temporal or spatial differences. To overcome these transportability problems, the NPI and Lpi were updated on half of our dataset (training set, see above) and validated on the remaining part (test set). The models were updated by model revision [12], which involves a re-estimation of the coefficients. As in [6], the updated prognostic indices are built from the resulting Cox proportional hazard regression model. 

**Figure 1:**
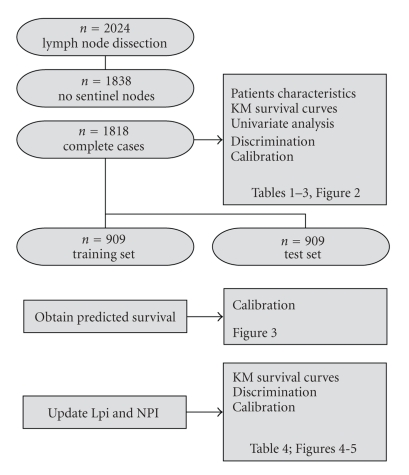
Flow chart: from the 2024 patients treated in our institution, 1,818 were eligible for this study. This cohort was used to summarize patient characteristics, Kaplan-Meier survival curves, and univariable survival analysis. The discriminating ability of the NPI and Lpi were compared on this cohort. Calibration of both models was checked on the test set (a random half of the data), after obtaining the predicted survival curves on the training set. The same training set was used to update both models. Kaplan-Meier curves, discrimination, and calibration ability of the updated models were calculated on the test set.

To categorize patients into risk groups, 1000 bootstraps of the training data set were used. In each bootstrap the lower and higher cutoff values were varied in steps of 0, 2. The pair leading to the largest cindex on most of the bootstraps was selected. Figure 1 gives a flow chart of the data and analysis flow. All statistical analyses were carried out using the software packages SAS 9.1.3 service pack 4, the level of significance being set at *α* = 0,05.

## 4. Results

### 4.1. Patient Characteristics

The median age at diagnosis was 57 years (IQR 48-67). All patients were treated with local surgery. The median number of dissected lymph nodes for all patients was 16 (IQR 12–21). A total of 38% (695/1818) of patients had lymph node metastasis with on average 4.11 (IQR 1–5) involved nodes. The median follow-up time was 5.92 year (IQR 4.33–7.12 year). Patients' characteristics are summarized in Table 1.

### 4.2. Discrimination and Calibration

**Figure 2:**
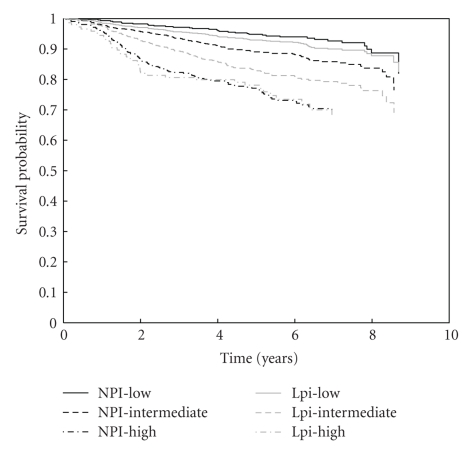
Kaplan-Meier survival curves for risk groups according to NPI (black lines) and Lpi (grey lines). The solid, dashed, and dot-dashed lines represent the low, intermediate, and high-risk group, respectively. The survival curves for both low-risk groups and for both high risk groups do not differ significantly. However, since the Lpi classifies significantly more patients into the low-risk group, the survival for Lpi intermediate risk patients is significantly lower than for NPI intermediate patients.

**Figure 3:**
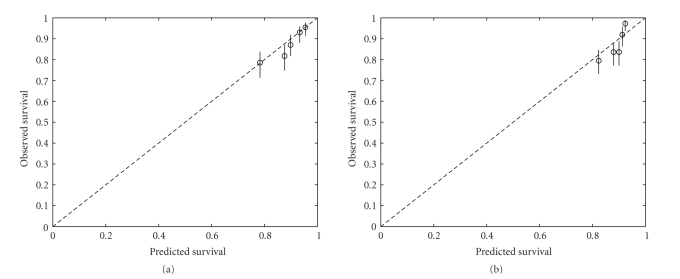
Calibration plot: (a) NPI, (b) Lpi. The test data are divided into 5 groups according to the value of the NPI (Lpi). For each group, the observed survival as calculated by the Kaplan-Meier estimator at 5 years and the median predicted survival at 5 years are plotted (circles). Ideally, the circles should lie on the dashed line. 95% conference intervals on the observed survival probabilities are represented by the vertical lines. The NPI is better calibrated than the Lpi.

Figure 2 illustrates the KM curves for NPI and Lpi risk groups. The number of patients in the low, intermediate, and high risk group for the NPI are 464, 934, 420, and for the Lpi 934, 736, 148, respectively. Although the Lpi has much more low risk patients than the NPI, DFS of both groups does not differ significantly (*P* =  .2176). Due to the allocation of patients to lower risk groups in the Lpi the DFS of the intermediate Lpi group is significantly lower than that for intermediate NPI patients (*P* < .001). As a first analysis, the significance of all variables in both the NPI and Lpi is checked in a univariate Cox model (Table 2). All variables included in both prognostic indices remain significant in our series. Table 3 summarizes the performance of both models. The NPI has a significantly higher cindex, but a significantly lower EXT%. The models do not differ in the difference between high and low risk event rates. Figure 3 shows that the NPI is better calibrated than the Lpi.

### 4.3. Model Updating

**Figure 4:**
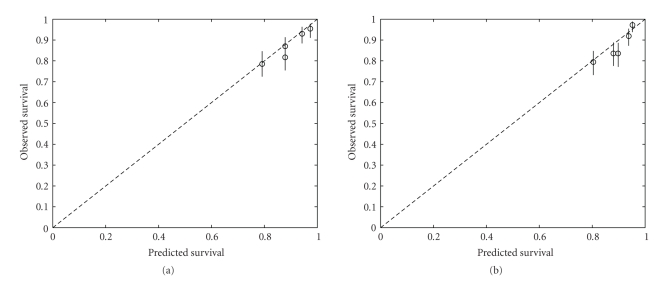
Calibration plot after updating the models: (a) NPI, (b) Lpi. The test data are divided into 5 groups according to the value of the NPI (Lpi). For each group, the observed survival as calculated by the Kaplan-Meier estimator at 5 years and the median predicted survival at 5 years are plotted (circles). Ideally, the circles should lie on the dashed line. 95% conference intervals on the observed survival probabilities are represented by the vertical lines. Both indices are well calibrated.

The major difference between the NPI and the Lpi lies in the substitution of staging of the number of positive lymph nodes by the ratio of positive versus negative nodes. In [13, 14], it was indicated that the ratio of positive versus the total number of examined lymph nodes is a better prognostic than the number of positive nodes per se. However, the number of examined nodes can differ between centres, which would result in different model coefficients. Therefore, both models are updated [12] using a random half of the dataset (training set). The updated NPI is built as 0.8xgrade + 0.4xnodal score, where grade and nodal score are defined as previously. The updated Lpi is built as 0.9 (if the grade is larger than 2, zero otherwise) + 0.4xlog ((npos + 0.5)/(nneg + 0.5)). The size was dropped from both formulas since the effect was no longer significant when taken together with the nodal score or lymph node ratio. Table 4 summarizes the performance of both updated models. No statistical differences are noted between both models. Figure 4 illustrates that both updated models are well calibrated.

**Figure 5:**
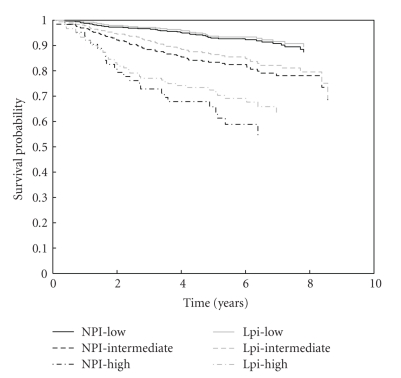
Kaplan-Meier survival curves for risks groups according to the NPI (black lines) and Lpi (grey lines). The solid, dashed, and dot-dashed lines represent the low, intermediate, and high risk group, respectively. The survival curves for corresponding NPI and Lpi groups do not differ significantly.

Using 1000 bootstrap samples of the training set, the optimal cutoffs for NPI and Lpi were obtained as {2.85, 3.65} and {−0.90, 0.00}, respectively. Figure 5 illustrates the KM survival estimates for the updated risk groups. Survival curves for corresponding NPI and Lpi groups did not differ significantly (*P* > .25). Comparing the original models with the updated models the concordance for the NPI was 0.93, whereas the concordance for the Lpi was 0.77. Since the updated NPI corresponds that well with the original NPI, the major difference is due to the change of the threshold values. For the Lpi, both the updating of the coefficients and the change in threshold leads to a better model.

## 5. Discussion

In our institution, the NPI is taken into account to estimate a patient's risk for relapse of breast cancer and to decide upon the modality of systemic therapy. The combined expression of ER, PR, and HER-2 is also taken into account, not only for prognostic but also for predictive purposes. This paper investigated the potential additional benefit of the number of involved lymph nodes or the lymph node ratio. 

Various prognostic factors have been described in breast cancer of which the axillary lymph node status and NPI are considered to be the most important ones. However, a tendency towards assessment of the nodal ratio can be seen with various studies indicating a better prognostic stratification of patients by nodal ratio than by number of positive lymph nodes [13–21]. Furthermore, an alternative nodal prognostic index for the established NPI has been put forward [8]. In the present study, we retrospectively reviewed all clinical charts and pathological reports from 1818 operable breast cancer patients which were treated in our institution between 2000 and 2005. We determined the NPI and Lpi value of all patients, updated NPI, and Lpi, categorized patients into risk groups and evaluated the results with regard to disease-free survival.

In contrast to the report of Vinh-Hung and colleagues, we did not find a significant improvement of the Lpi above the NPI, although we previously confirmed the superiority of the LNR above the number of positive lymph nodes [14]. After updating both models, this conclusion remained. Since no significant differences between the Lpi and the NPI are noted, the NPI is preferred above the Lpi. The former index is less dependent on centre since it only includes well-defined variables. The Lpi on the contrary includes the number of examined lymph nodes which might vary according to the centre/surgeon. Therefore, Lpi would need updating to acquire the same performance as the NPI. 

We believe this study to be of great value despite its smaller size in comparison with the study of [8]. First, our patient population is representative for the overall breast cancer population with respect to the median age at diagnosis and number of patients in the various NPI risk groups [22, 23]. Second, all patients in the study were diagnosed and treated in a single institution by one multidisciplinary team. However, our study also has its weaknesses such as the lack of overall survival analyses and a relatively short follow-up time.

## 6. Conclusion

This study aimed at evaluating the prognostic value of the Lpi with respect to disease-free survival. However, besides the Lpi various other prognostic indices are under investigation as improvement of the established NPI [22, 24]. Based on the results from our study, we can conclude that the Lpi does not perform better than the NPI. 

## Figures and Tables

**Table 1:** Patients' characteristics.

Characteristic	No. of Patients (*n* = 1818)	%
Age, years		
Median	57	
Lower-upper quartiles	48–67	

Histologic grade		
low	242	13.31
intermediate	823	45.27
high	753	41.42

Tumor size, cm		
Median	2.3	
Lower-upper quartiles	1.5–3.5	

No. of lymph nodes removed		
Median	16	
Lower-upper quartiles	12–21	

No. of positive lymph nodes		
Median	0	
Lower-upper quartiles	0-1	
Range	0–42	

Pathologic nodal stage (pN)		
0	1123	61.77
1–3	456	25.08
>3	239	13.15

Node ratio		
Median	0.04	
Lower-upper quartiles	0.03–0.14	

Adjuvant treatment		
No adjuvant treatment	37	0.02
Radiotherapy	1519	83.55
Chemotherapy	655	36.03
Endocrine Therapy	1430	78.66

**Table 2:** Cox univariate analysis of prognostic variables included in the NPI and Lpi, and NPI and Lpi.

Variable	HR	95% CI	*P*-value
Size (cm)	1.081	1.028–1.136	.0023
pN			
low versus high	0.527	0.453–0.612	<.0001
pN			
intermediate versus high	0.630	0.512–0.775	<.0001
Grade			
low versus high	0.432	0.350–0.533	<.0001
Grade			
intermediate versus high	0.642	0.559–0.737	<.0001
Lymph node ratio	1.427	1.328–1.533	<.0001
Grade			
low and intermediate versus high	2.644	2.057–3.398	<.0001

HR: hazard ratio

pN: pathological nodal status

**Table 3:** Measures of model performance of NPI and Lpi. Significant better performance is indicated in bold.

measure	NPI	Lpi	95% CI for the difference between NPI and Lpi
cindex	**0.69**	0.66	0.01; 0.04
EvR	0.04	0.04	−0.01; 0.02
EXT%	48.6	**59.5**	−0.14; −0.08

EvR: difference in event rate in high versus low risk patients

EXT%: percentage of patients classified into the most extreme risk groups

CI: confidence interval.

**Table 4:** Measures of model performance of NPI and Lpi, after updating. Significant better performance is indicated in bold.

measure	NPI	Lpi	95% CI for the difference between NPI and Lpi
cindex	0.69	0.69	−0.02; 0.02
EvR	0.08	0.05	−0.00; 0.05
EXT%	0.66	0.67	−0.04; 0.01

Difference in event rate in high versus low risk patients

EXT%: percentage of patients classified into the most extreme risk groups

CI: confidence interval.
